# Atypical course in severe catatonic schizophrenia in a cannabis-dependent male adolescent: a case report

**DOI:** 10.1186/s13256-015-0678-5

**Published:** 2015-09-21

**Authors:** Anders Håkansson, Björn Axel Johansson

**Affiliations:** 1 Department of Clinical Sciences Lund, Division of Psychiatry, Lund University, Malmö Addiction Centre, Södra Förstadsgatan 35, Malmö, S-205 02 Sweden; 2 Department of Clinical Sciences Lund, Division of Child & Adolescent Psychiatry, Lund University, Malmö, Sweden; 3 Office for Healthcare ‘Sund’, Child & Adolescent Psychiatry, Regional Inpatient Care, Emergency Unit, Malmö, Sweden

## Abstract

**Introduction:**

Adolescents with psychoses usually have full recovery from their first psychotic episode, but the first relapse often arises within 2 years of the first episode. Cannabis-related psychoses are difficult to distinguish from schizophrenic psychoses. Here, we describe a particularly severe clinical case, with a first psychotic episode occurring after heavy cannabis smoking, an atypically long symptom-free duration, and a subsequent non-substance-related episode.

**Case presentation:**

A 17-year-old male adolescent of Middle-East origin presented with delusions and hallucinations after extensive cannabis smoking. His first psychotic episode, with paranoid delusions and hallucinations, progressed into severe catatonic symptoms. His symptoms were treated with electroconvulsive therapy and risperidone and he was transferred to a residential substance abuse treatment center. He remained drug-free and non-psychotic for 3.5 years. Given the temporal association with extensive cannabis use, and his full remission of symptoms lasting several years, a cannabis-induced psychosis—though atypically extended—could be suspected. However, after 3.5 years without psychiatric care, and in a drug-free state, our patient again presented with positive psychotic symptoms, possibly induced by a period of severe psychosocial stress.

**Conclusion:**

We here discuss whether a primary schizophrenic episode possibly induced by cannabis can increase the risk of subsequent non-drug-related schizophrenic episodes.

## Introduction

Adolescent schizophrenia, in comparison to the adult-onset forms, is associated with an increased family history of schizophrenia; male predominance; more premorbid anomalies in speech, psychomotor, and social development; intelligence below average; insidious onset; fewer systematic delusions and hallucinations [1–3]; and more negative symptoms such as flattened affects and bizarre behavior [1]. It can present with enuresis and incontinence during psychosis [4]. The distinction between adolescent schizophrenia and other diagnoses, such as affective psychosis, may be difficult [1]. Adolescents with schizophrenia usually make a full recovery after the first psychotic episode, but after 6 months of psychosis, the prognosis for full recovery is poor [1]. The first relapse often arises within 2 years of the end of the first episode [5–7]. Several potential predictors of relapse have been discussed, including rearing problems in the family, ethnicity, migrant status, urban upbringing [8], treatment discontinuation [7], social adjustment, and poor adaptation to school [6].

An acute and usually transient psychosis, including a confusional condition with delusions or hallucinations, has been associated with prolonged cannabis intoxication [9, 10]. Cannabis-related psychoses may be difficult to distinguish from schizophrenic psychoses [11], but may be partly distinguishable [12, 13] by a shorter duration than schizophrenic episodes (typically from hours to a few weeks) and, typically, an onset after intense cannabis smoking. Patients generally remain non-psychotic in the absence of drug relapse [13, 14].

Here, we describe a severe clinical case of catatonic schizophrenia in a male adolescent with a history of substantial cannabis smoking, and with an atypically long period of remission between the first and second psychotic episodes. Our aim in presenting this case is to demonstrate the diagnostic challenge in cannabis-induced psychosis vis-à-vis schizophrenia, and to demonstrate the atypical course of a patient with a psychotic disorder but with a particularly long symptom-free period between his first and second psychotic episodes. We also highlight the discussion about whether cannabis might potentially increase the risk of subsequent non-cannabis-related psychotic episodes.

## Case presentation

Six years ago, a 17-year old adolescent male of Middle-East origin was brought to our emergency room at the Department of Child & Adolescent Psychiatry, Malmö, Sweden, by his mother and stepfather. Over the previous weeks, he had reported being observed by authorities and criminal gangs. He talked to himself, felt that TV broadcasts referred to his mother, and had aggressive outbursts.

Our patient lived with his mother, stepfather, and a 15-year-old brother. He had no family history of psychiatric disease. His psychomotor development had been normal. After his parents’ divorce, our patient moved from a Middle-Eastern country to Sweden with his mother and brother. He started to use illicit drugs before age 13. Contacts with social authorities and a regional addiction center were established.

In our emergency room, our patient’s mother reported that he had been smoking cannabis daily for 4 years, up to 3g per day. For the past 2 years, he had also misused tramadol, taking 200–1,000mg daily. He had tried lysergic acid diethylamide, cocaine, hallucinogenic mushrooms, ecstasy, inhalants, and different analgesics. For the 3 weeks preceding the emergency room visit, he had abstained from cannabis and tramadol after being enrolled in a youth service program related to a conviction for robbery and cannabis possession. Five days prior to admission, he still screened positive for tetrahydrocannabinol (THC). Upon examination, our patient was mute, reacted inappropriately, and presented a depressed mood. A urine toxicology test was negative for THC and other substances. After evaluation, he returned home with a short-term prescription of one antipsychotic medication in a very low dose (levomepromazine, 10mg at night, for 2 consecutive days) and alimemazine (20mg as needed, for 2 days), and with a short-term outpatient appointment.

Six days later, still with pronounced ideas of reference, he was voluntarily admitted to the Addiction Centre of our hospital. A urine toxicology test was still negative. After 4 days of inpatient care with persistent delusions, he was transferred to the Department of Child & Adolescent Psychiatry. He reported feelings of being observed by staff and by perceived cameras in street lights, and he laughed without obvious reason. Results from a blood screen, computed tomography of his brain, and an electroencephalogram (EEG) were normal. Our patient’s delusions and hallucinations gradually diminished. After 10 days of inpatient care without specific psychopharmacological treatment, he communicated almost adequately and was discharged to a residential addiction treatment center.

One week after his arrival at the treatment institution, our patient’s behavior was again altered. He was assessed at the local Department of Child & Adolescent Psychiatry, where he isolated himself, and presented bizarre body postures and inappropriate laughs. A urine toxicology test was negative for THC and other drugs. His arm appeared to be paralyzed and he collected saliva in his mouth. After consultation with pediatric expertise, he was transferred back to the Department of Child & Adolescent Psychiatry in his hometown, after receiving an intramuscular injection of 10mg of olanzapine.

Upon arrival, he was mute and sat completely still, eyes closed, with saliva running down his chin. He also presented with urine and fecal incontinence, and with asymmetric convulsions in his arms and legs. His blood pressure (130/60mmHg) and heart rate (80 beats per minute) were normal. Results of an EEG and magnetic resonance imaging (MRI) of his brain were normal. A pediatric neurologist did not find signs of physical illness. Possible differential diagnoses included substance-induced psychosis, depressive stupor, catatonic schizophrenia and neuroleptic malignant syndrome (NMS). A nasogastric tube was introduced for nutrition. He received 20mg of diazepam daily. Electroconvulsive therapy (ECT) was initiated along with risperidone, initially 1mg per day. During the next 5 weeks, he received ECT 12 times with bilateral stimulation. Risperidone was gradually increased to 3mg per day. After the seventh ECT treatment, he opened his eyes and started to communicate with his hands. After the eighth ECT, the nasogastric tube was withdrawn and the diazepam dose was reduced by half. Occasionally he talked about the mafia, laughed inappropriately, and expressed doubts about his medication. After the ninth ECT, the risperidone dose was increased to 4mg per day, and our patient gradually improved. Colleagues from the outpatient Psychosis Team confirmed the preliminary diagnosis of catatonic schizophrenia. Our patient fulfilled diagnostic criteria of catatonic schizophrenia according to the International Classification of Diseases 10th Revision (ICD-10 [15]), including the general schizophrenia criterion (criterion A), at least four out of seven cluster B criteria (criteria number 1, 3, 4, and 5), and the C criterion excluding another factor likely to cause catatonic behavior. Thus, he was diagnosed with catatonic schizophrenia (F20.2) and cannabis dependence (F12.2) upon discharge from inpatient treatment, with a prompt appointment with the Psychosis Team.

During the first few weeks after discharge, our patient relapsed into tramadol and cannabis abuse. Paranoid delusions and hallucinations re-appeared, and 4 months after discharge he was re-hospitalized. Symptoms improved and he was successfully discharged to another residential addiction treatment institution.

Five years ago, having been abstinent from illicit drugs for almost 4 months, our patient started work. The follow-up contact with the Psychosis Team was limited to occasional telephone appointments and finally ceased. Our patient met and moved in with a girlfriend. He started formal training to become a nursing assistant, and maintained good relationships with his girlfriend and friends.

Two years ago, our patient started to feel stressed; his girlfriend’s parents expressed doubts about his employment, and he worked and studied at the same time. He started to be absent from classes and suffered from insomnia and weight loss. When his girlfriend traveled, he became suspicious, developed delusions about being poisoned, and changed to vegetarian food. When his girlfriend returned home, he was irritable and expressed ideas of references related to TV broadcasts.

Our patient started to spend time with his mother, who reported that he occasionally presented bizarre postures and screamed without reason. On one occasion, he also behaved aggressively towards his mother, and held a knife against his own neck. He was transported by the police to the psychiatric emergency room, where he presented with bizarre facial expressions, inappropriate laughs, paranoid and bizarre delusions, and aggressive and disrupted behavior. Results from a urine toxicology test were negative. He was admitted for compulsory psychiatric treatment, including temporary physical restraint and acute intramuscular treatment with zuclopenthixol. Subsequently, his medication was changed to risperidone in doses increasing to 6mg per day per os. Our patient’s mother was opposed to the medication. After two weeks of inpatient treatment he was discharged in a somewhat improved condition.

Owing to anxiety and sleeping problems, our patient returned to the psychiatric emergency unit on several occasions after discharge. He was prescribed levomepromazine without significant effect. One week after discharge, he was brought to hospital after an overdose with a prescribed medication, reportedly levomepromazine. He denied any suicidal intent and his urine toxicology screen was negative. Our patient was again hospitalized for compulsory psychiatric treatment. He was paranoid and dysphoric. Again, his mother was opposed to pharmacological treatment. After 5 days he was discharged with a prescription for risperidone, 25mg weekly, to be administered via the intramuscular route by the Psychosis Team.

During the next 4 months, he was seen by a senior consultant at the Psychosis Team on five occasions. Initially he presented with a somewhat dysphoric mood, and inadequate smiles were reported. He was continuously opposed to the medication, and requested a change from injections to oral medication. He started treatment with risperidone, 2mg daily per os. At follow-up, 1 year ago, he reported taking his medication and felt well.

## Discussion

### Differential diagnoses

Our experience with this case illustrates that adolescent schizophrenia can be challenging to classify in individuals with a history of smoking cannabis. The onset was acute with delusions and hallucinations, supporting a cannabis-related or possibly affective psychosis. Owing to the clinical picture, our patient was diagnosed with schizophrenia despite a history of heavy cannabis abuse. A catatonic subtype was determined given the motor immobility, extreme negativism and mutism, and bizarre postures and facial expressions [15]. NMS was ruled out because changes in his level of consciousness, muscular rigidity (bizarre body postures), and autonomic instability (urine incontinence and salivation) began before therapeutic doses of antipsychotic medication were prescribed. The absence of leukocytosis, hyperthermia, and tachycardia along with a normal EEG supports other diagnostic alternatives [16]. After 3.5 years of social adjustment without psychiatric treatment, our patient’s first psychotic episode was more likely to be classified as cannabis-induced, despite the atypically pronounced duration and severity of that first episode [13, 14]. However, after relapsing into pronounced psychotic symptoms without any signs of substance abuse, he was re-diagnosed with catatonic schizophrenia.

The temporal association of our patient’s first psychotic episode with heavy cannabis use clearly raises questions about a potential role of cannabis in psychotic disorders. There is a relatively large amount of literature describing the association between cannabis and acute and chronic psychosis [9–14, 17–19]. Here, the onset of our patient’s psychosis was acute rather than insidious, typically suggesting a cannabis-induced psychosis rather than schizophrenia, and although results of his urine toxicology had returned to normal after 3 weeks of social authority supervision, the temporal association with heavy cannabis consumption was confirmed by our patient’s family.

The acute onset with positive symptoms, along with a nearly full remission after 10 days of inpatient care, could have pointed towards an affective psychosis [4]. However, our patient presented no signs of elevated mood. Depressive stupor was considered; our patient presented with depressive symptoms, possibly mood-congruent delusions, and he was clearly improved by ECT, but severe psychotic symptoms including motor and vegetative symptoms clearly dominated the clinical picture, and an affective diagnosis was rejected in favor of catatonic schizophrenia [20, 21]. Normal blood test results, an MRI of his brain, an EEG, and consultation with pediatric expertise ruled out neurodegenerative psychosis. Thus, after an initial interpretation of the symptoms as manifestations of a cannabis-induced psychosis, a primary catatonic schizophrenia appeared a considerably more likely explanation. While an entirely substance-related etiology was ruled out, we cannot exclude that cannabis played a significant role in the onset of the first episode, and that it may even have predisposed our patient to a subsequent psychotic episode, potentially precipitated by a stressful situation. Although the literature is not conclusive in this area, it cannot be ruled out that heavy cannabis abuse can be a predictor of later psychosis [18].

### Proximal stressors

Our experience in this case suggests that adolescent schizophrenic episodes in the same patient may potentially be elicited by different stressors, for example, cannabis smoking [17–19] and psychological stress [22, 23], and cause almost the same clinical presentation. This case illustrates the potential role of proximal risk factors for the development of schizophrenic episodes [17–19, 22, 23]. Our patient had no known heredity predisposition for schizophrenia, but was vulnerable in other aspects, including stressful life events. He was brought up in a war-torn country, his parents were divorced, and he became a migrant with an urban up-bringing at the age of 12 years. Soon after arriving in Sweden he became socially marginalized and failed academically. It cannot be excluded that these circumstances, together with heavy cannabis smoking, contributed to his illness [8, 17, 18].

### Relapse prevention

An asymptomatic duration of 3.5 years of social adjustment, without medication and psychiatric services, is uncommon after the first severe schizophrenic episode [5–7], making the clinical course of the present case unusual. Continued contact with the Psychosis Team probably would have reduced the risk for relapse [1, 23]. The change from a hazardous lifestyle with heavy cannabis smoking to a drug-free state with social adaption was not enough for our patient to maintain health. With increased social adjustment, our patient might have experienced a feeling of false safety, making him end the outpatient contact with the Psychosis Team. This case emphasizes the importance of educating a patient and their parents, especially in cases where there is hesitation about treatment, because compliance is likely to be fundamental in the control of symptoms [1, 24]. Our patient’s mother was critical to the pharmacological treatment, and this may have contributed to the short treatment period and re-hospitalization one week after discharge [25]. Pharmacological treatment is crucial for recovery [7], and more psycho-educational efforts could have been made in the communication with our patient and his parents, and potentially could have improved compliance with treatment [1]. Finally, this case demonstrates the importance of maintaining at least a sparse outpatient contact after a first psychotic episode, although this may be challenging in patients who are currently not presenting subjective symptoms of disease [1, 6, 24].

## Conclusions

Diagnostic classification of adolescents with psychotic symptoms can be challenging. Here, an assumingly cannabis-induced psychotic episode was followed by a 3.5-year symptom-free period in absence of drugs, but pronounced psychotic symptoms re-appeared in the absence of drugs. We highlight the question of whether a primary psychosis induced by cannabis can increase the risk of subsequent non-drug-related psychoses.

## Consent

Written informed consent was obtained from the patient for publication of this case report. A copy of the written consent is available for review by the Editor-in-Chief of this journal.

## Abbreviations

ECT: electroconvulsive therapy
EEG: electroencephalogram
MRI: magnetic resonance imaging
NMS: neuroleptic malignant syndrome
THC: tetrahydrocannabinol
